# Terpinen-4-ol Induces Apoptosis in Human Nonsmall Cell Lung Cancer In Vitro and In Vivo

**DOI:** 10.1155/2012/818261

**Published:** 2011-06-20

**Authors:** Chieh-Shan Wu, Yun-Ju Chen, Jeremy J. W. Chen, Jeng-Jer Shieh, Chia-Hsin Huang, Pei-Shan Lin, Gee-Chen Chang, JingHua-Tsai Chang, Chi-Chen Lin

**Affiliations:** ^1^Department of Dermatology, School of Medicine, College of Medicine, Kaohsiung Medical University, Kaohsiung 80708, Taiwan; ^2^Department of Child Care, College of Humanities and Social Sciences, Southern Taiwan University, Tainan 71005, Taiwan; ^3^Institute of Biomedical Sciences, College of Life Science, National Chung Hsing University, Taichung 40227, Taiwan; ^4^Agricultural Research Institute, Council of Agriculture Executive, Yuan 10014, Taiwan; ^5^Department of Internal Medicine, Division of Chest Medicine, Taichung Veterans General Hospital, Taichung 40705, Taiwan; ^6^Department of Medical Education and Research, Taichung-Veterans General Hospital, Taichung 40705, Taiwan; ^7^Institute of Medicine, Chung Shan Medical University, Taichung 40201, Taiwan

## Abstract

Terpinen-4-ol, a monoterpene component of the essential oils of several aromatic plants, exhibits antitumor effects. In this study, the antitumor effects of terpinen-4-ol and the cellular and molecular mechanisms responsible for it were evaluated and studied, respectively on human nonsmall cell lung cancer (NSCLC) cells. Our results indicated that terpinen-4-ol elicited a dose-dependent cytotoxic effect, as determined by MTT assay. Increased sub-G1 population and annexin-V binding, activation of caspases 9 and 3, cleavage of poly(ADPribose) polymerase (PARP), and a decrease of mitochondrial membrane potential (MMP) indicated involvement of the mitochondrial apoptotic pathway in terpinen-4-ol-treated A549 and CL1-0 cells. Elevation of the Bax/Bcl-2 ratio and a decrease in IAP family proteins XIAP and survivin were also observed following terpinen-4-ol treatment. Notably, terpinen-4-ol was able to increase p53 levels in A549 and CL1-0 cells. Diminution of p53 by RNA interference induced necrosis instead of apoptosis in A549 cells following terpinen-4-ol treatment, indicating that terpinen-4-ol-elicited apoptosis is p53-dependent. Moreover, intratumoral administration of terpinen-4-ol significantly suppressed the growth of s.c. A549 xenografts by inducing apoptosis, as confirmed by TUNEL assay. Collectively, these data provide insight into the molecular mechanisms underlying terpinen-4-ol-induced apoptosis in NSCLC cells, rendering this compound a potential anticancer drug for NSCLC.

## 1. Introduction

Lung cancer is the leading cause of cancer-related deaths worldwide. Among lung cancers, nonsmall cell lung carcinomas (NSCLC) account for approximately 80% of lung cancer cases [1]. Despite improvements in survival through early detection and treatment, rapid disease recurrence and progression still plague some patients [2]. Thus, the search for new therapeutic approaches is still important and urgently needed in clinical oncology.

Monoterpenes are major plant-derived secondary metabolites; they consist of two isoprene units, are found in essential oils, and are associated with plant defense [3, 4]. In addition, numerous monoterpenes have been proposed to exert potent antitumor action, and some have shown promising results in the prevention and treatment of a variety of cancers in tumor model systems [5, 6]. Notably, two naturally occurring monoterpenes, perillyl alcohol (POH) and limonene (LIM), are currently undergoing clinical trials to evaluate their therapeutic effect [7, 8]. 

Terpinen-4-ol, a naturally occurring monoterpene found in the essential oils of many aromatic plants including Melaleuca alternifolia (tea tree oil), Hajeb Layoun arboreta (Tunisia) and Alpinia zerumbet, has been shown to have antiviral, antibacterial, antifungal, and insecticidal effects as well as antioxidant and anti-inflammatory activities [9–13]. Recent reports have indicated that terpinen-4-ol exerts its antitumor effects by triggering caspase-dependent apoptosis in human melanoma cells or by inducing necrotic cell death and cell-cycle arrest in mouse mesothelioma and melanoma cell lines without affecting normal cells [14, 15]. 

Although these findings demonstrate the anticancer activity of terpinen-4-ol, the underlying molecular mechanisms of the antitumor activity of terpinen-4-ol remain unclear. In addition, there is no report on the antitumor effects of terpinen-4-ol against human nonsmall cell lung cancer cells. Therefore, in this study, the anticancer effects of terpinen-4-ol were evaluated on two NSCLC cell lines, namely, A549 and CL1-0 human lung adenocarcinoma cells. The possible molecular mechanisms responsible for its anticancer activity were also investigated. Our results indicated that terpinen-4-ol induced apoptosis through a mitochondria-mediated pathway in NSCLC cells and that the apoptosis elicited by terpinen-4-ol was p53 dependent. Furthermore, treatment of s.c xenografts derived from A549 cells with intratumor injections of terpinen-4-ol significantly inhibited tumor growth compared with the control group.

## 2. Materials and Methods

### 2.1. Cell Culture and Reagents

The A549 human lung adenocarcinoma and CL1-0 lung adenocarcinoma cell lines were cultured in Dulbecco′s modified eagle medium supplemented with 10% fetal bovine serum (FBS) and 1% antibiotic antimycotic. Cultures were maintained in a humidified incubator with 5% CO_2_ at 37°C. The A549/p53-shRNA clone 14 cells were established in culture as described by Chang et al. [16]. Terpinen-4-ol (Sigma-Aldrich, St. Louis, MO) was 97% pure. A 0.2% stock solution of terpinen-4-ol was prepared and was subsequently diluted to 0.02%–0.1% in warm supplemented media [14].

### 2.2. Cytotoxicity Assay

The cytotoxic effects of terpinen-4-ol on A549 and CL1-0 cells were measured with the 3-[4,5-dimethylthiazol-2-yl]-2,5 diphenyltetrazolium (MTT) assay (Sigma-Aldrich, St. Louis, Mo, USA). The A549 and CL1-0 cells were seeded onto 24-well plates for 24 hours. Various concentrations of terpinen-4-ol were added to the cells. After incubation for 24 hours, the medium was removed, and 200 *μ*L of 1 × MTT solution was added to each well for 4 hours. The spectrophotometric absorbance of each well was analyzed by a microplate reader (TECAN) at 540 nm. For analysis of the cytotoxic efficiency, we calculated IC_50_ values with dose-dependent curves by linear interpolation.

### 2.3. Western Blot Analysis

Whole-cell protein was lysed with 2% SDS (10 mM EDTA, 50 mM Tris base, 10% SDS, pH 8.0) and boiled at 100°C for 10 minutes. Protein concentrations were measured using the BCA protein assay. Equal amounts of protein were loaded on 10%–15% SDS-PAGE gels, transferred to PVDF membranes and blocked with 5% nonfat milk in TBST buffer (20 mM Tris-HCl, 120 mM NaCl, 0.1% Tween 20) for 1 hour. Membranes were incubated with various primary antibodies against Cyclin B1, Cdc2, Cdc25c, Bcl-2, Bax, caspase-3, caspase-9, and PARP at 4°C overnight. After washing, the blots were incubated with HRP-labeled secondary antibodies for 2 hours. The signals of the blots were then developed using the enhanced chemiluminescence (ECL) system and analyzed with the LAS3000 system (Fujifilm, Tokyo, Japan).

### 2.4. Mitochondria/Cytosol Fractionation

A mitochondria/cytosol fractionation kit (Biovision) was used for isolation of the mitochondrial fraction from the cytosolic fraction of mammalian cells. Cells were harvested by trypsinization, washed twice with PBS and resuspended in 500 *μ*L of cytosol extraction buffer mix on ice for 10 minutes. Cells were homogenized with a Dounce tissue grinder (Biovision) on ice for 30–50 passes and centrifuged at 3000 rpm for 10 minutes at 4°C. The supernatants were collected and centrifuged again at 13000 rpm for 30 minutes at 4°C.

### 2.5. Cell Cycle Analysis and Sub-G_1_ Measurement

3 × 10^5^ cells were plated on 6 cm dishes for 24 hours. The cells were then treated with different concentrations of terpinen-4-ol for different periods of time, followed by collecting the cells by centrifugation. The pellets were mixed with 75% ethanol at −20°C overnight. The cells were then centrifuged and resuspended in 500 *μ*L of PI staining solution (2 mg/mL RNase, 1 mg/mL PI, 5% Triton X-100) for 1 hour at 37°C in the dark. The cells were next analyzed by FACS Calibur flow cytometry (BD Biosciences). The distribution and percentages of cells in the sub-G1, G0/G1, S, and G2/M phases of the cell cycle were further analyzed with WinMDI software (version 2.9, Joseph Trotter).

### 2.6. Annexin V Assay

The BioVision annexin V-FITC apoptosis detection kit was used for the apoptosis assay (BioVision). A549 and CL1-0 cells were seeded onto 6 cm dishes for 24 hours and then exposed to different doses of terpinen-4-ol for 6 hours. Cells were harvested by trypsinization, washed twice with PBS, and resuspended in 500 *μ*L of binding buffer. Cell suspensions were then incubated with 5 *μ*L of annexin V-FITC and 5 *μ*L of propidium iodide (PI) for 10 minutes at room temperature in the dark. The cells were evaluated immediately by flow cytometry.

### 2.7. Mitochondrial Membrane Potential Assay

Mitochondria-specific cationic dye JC-1 (Invitrogen, Carlsbad, Calf, USA), which undergoes potential-dependent accumulation in the mitochondria, was used. When the membrane potential (ΔΨ) is below 120 mV, JC-1 is monomeric and emits green light (540 nm) following excitation with blue light (490 nm). At membrane potentials higher than 120 mV, JC-1 monomer aggregates and emits red light (590 nm) following excitation with green light (540 nm). For the assay, cells were seeded onto 96-well plates and treated with various concentrations of terpinen-4-ol for 24 hours, followed by staining with 25 *μ*M JC-1 for 30 minutes at 37°C. Fluorescence was monitored with a fluorescence plate reader at wavelengths of 490 nm (excitation)/540 nm (emission) and 540 nm (excitation)/590 nm (emission). Changes in the mitochondrial membrane potential were indicated by changes in the ratio of intensities between the measurements at test wavelengths of 590 nm (red) and 540 nm (green).

### 2.8. In Vivo Antitumor Activity

In total, 1 × 10^7^ A549 cells were s.c. injected into the right flank of 16 week old female BALB/c *nu*/*nu* mice. Tumor-bearing mice were subdivided into groups of five mice. Therapy was initiated 10 days after tumor inoculation when the mean tumor volume was 50 mm^3^. A stock solution of terpinen-4-ol was made by dissolving 11 *μ*L in 5.5 mL PBS with vigorous vortexing. This 0.2% solution was further diluted in PBS. Tween 80 (final concentration 0.001% v/v) was included to facilitate oil solubility [17]. Mice were intravenously infused daily with 100 *μ*L of terpinen-4-ol at the indicated doses (0.06% and 0.1%). Control mice were intravenously infused daily with 100 *μ*L of PBS containing 0.001% Tween 80. Tumor volumes were calculated as length × width × thickness × 0.5 and expressed in mm^3^.

### 2.9. TUNEL Assay

Apoptotic cell death in paraffin-embedded tumor tissue sections was examined using the TdT-FragEL DNA fragmentation detection kit (Calbiochem, San Diego, Calf, USA) according to the manufacturer's instructions. Apoptotic cells were identified as dark brown nuclei under a light microscope. The number of apoptotic cells was counted in five randomly selected high-power fields in a blinded manner.

### 2.10. Statistical Analysis

Results are expressed as mean ± SD. Statistical comparisons between two groups of data were made using an unpaired two-tailed Student's *t*-test, and *P* values <.05 were considered significant.

## 3. Results

### 3.1. Cytotoxic Effects of Terpinen-4-ol in A549 and CL1-0 Cells

**Figure 1:**
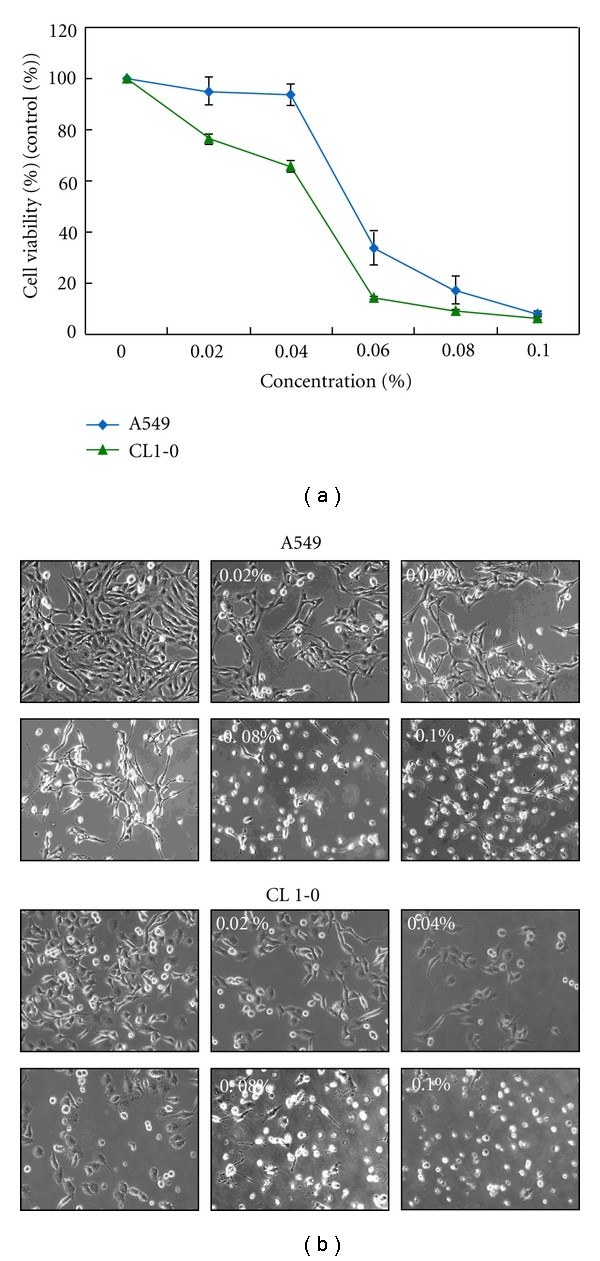
Effects of terpinen-4-ol on viability and morphology of A549 and CL1-0 cells. (a) A549 and CL1-0 cells were seeded at 5 × 10^4^ cells/well and then treated with the indicated concentrations of terpinen-4-ol for 24 hours following attachment. The cell viability was determined by the MTT assay. Each point on the graph represents the mean ± SD of triplicate tests. *P value <.05 compared with the untreated control group. (b) The morphology of A549 and CL1-0 cells treated with various concentrations of terpinen-4-ol for 24 hours were observed by phase-contrast microscopy and photographed (400×).

To determine the cytotoxic effect of terpinen-4-ol on cell, A549 and CL1-0 cells were treated with 0.02% to 0.1% terpinen-4-ol for 24 hours, and cell viability was determined using the MTT assay. As shown in Figure 1(a), the viability of A549 and CL1-0 cells was markedly reduced by terpinen-4-ol in a concentration-dependent manner. The results were expressed as a percentage relative to the control group. At 24 hours, the estimated IC_50_ values were 0.052% in A549 cells and 0.046% in CL1-0 cells, respectively. In addition, the morphological changes in terpinen-4-ol-treated cells at 24 hours were also examined using a phase contrast microscope, as illustrated in Figure 1(b). At concentrations up to 0.06%, cell proliferation was inhibited, but the cells remained mostly alive. At concentrations of 0.08% and higher, the compound induced cell death with marked morphologic changes such as shrinkage, rounding, and floating of the cells. These morphological changes suggested that terpinen-4-ol might induce apoptotic cell death in human NSCLC cells.

### 3.2. Terpinen-4-ol Induces Apoptosis in A549 and CL1-0 Cells

**Figure 2:**
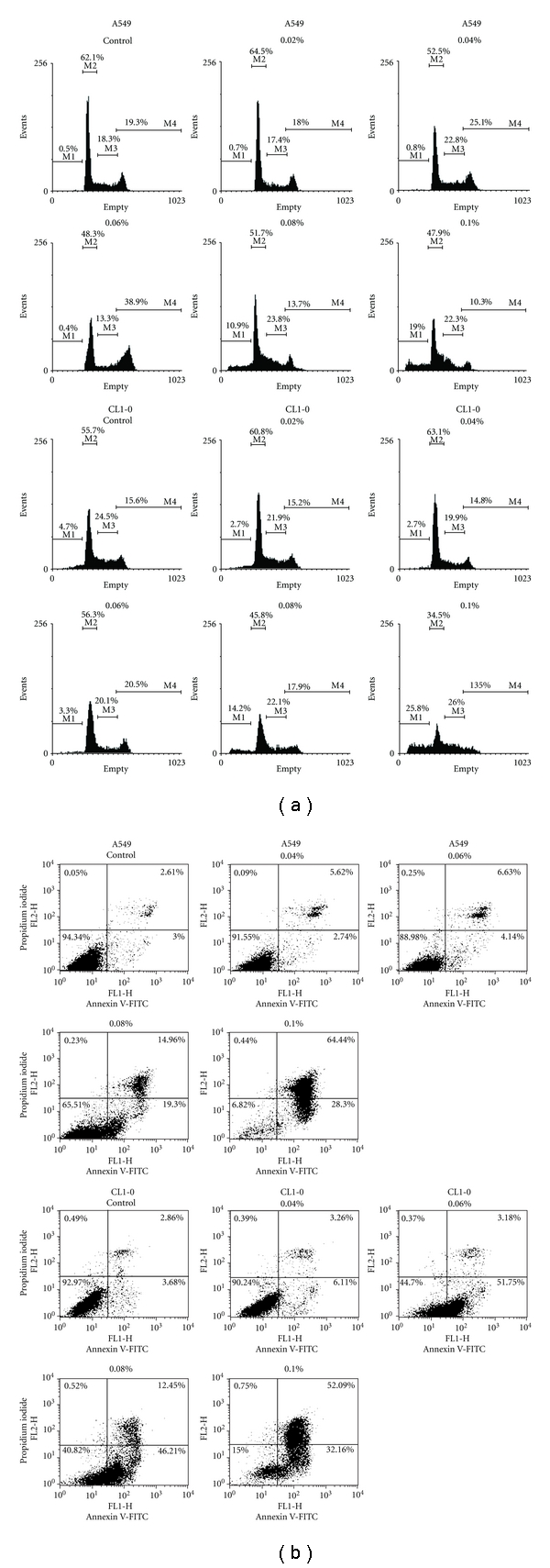
Effects of terpinen-4-ol on cell-cycle distribution in A549 and CL1-0 cells. (a) Cell-cycle analysis of terpinen-4-ol-treated cells. Cells were treated with the indicated concentrations of terpinen-4-ol for 24 hours, and then, cell-cycle distributions were determined by propidium iodide (PI) staining and subsequent flow cytometry analysis (Gating for each cell-cycle phase M1: <2N; M2: G0-G1; M3: S; M4: G2-M). Data are representative of three independent experiments with similar results. (b) Flow cytometry analysis of terpinen-4-ol-induced apoptosis in A549 and CL1-0 cells. The cells were treated with the indicated concentrations of terpinen-4-ol for 24 hours, followed by labeling for phosphatidylserine externalization with FITC-annexin-V and cell membrane integrity with PI. The lower right quadrant (annexin-V+/PI-) represents early apoptosis, while the upper right quadrant (annexin V+/PI+) represents late apoptosis and necrosis. Data are representative of three independent experiments with similar results.

To determine whether terpinen-4-ol causes cell growth inhibition by inducing cell-cycle arrest, A549 and CL1-0 cells were subjected to flow cytometric analysis. Both A549 and CL1-0 cells were exposed to a series of concentrations of terpinen-4-ol for 24 hours.  Figure 2(a) shows that when exposed to 0.06% terpinen-4-ol for 24 hours, a high number of cells were arrested in the G_2_/M phase. Additionally, the sub-G_1_ population, which is an indication of cell death, increased significantly in the presence of 0.08% and 0.1% terpinen-4-ol. To clarify the type of cell death elicited by terpinen-4-ol, A549 and CL1-0 cells were treated with various concentrations of terpinen-4-ol for 24 hours and subjected to flow cytometry analysis after staining with annexin V-FITC and propidium iodide (PI). As shown in Figure 2(b), the percentages of early apoptotic death (annexin V+/PI− and lower right quadrant) and late apoptotic and necrotic death (annexin V+/PI+ and upper right quadrant) increased in a dose-dependent manner in A549 cells. The percentage of early apoptotic death in CL1-0 cells rose at lower doses of terpinen-4-ol but fell slightly at higher doses, whereas the percentages of late apoptotic and necrotic death in CL1-0 cells still increased in a dose-dependent manner. The former phenomenon seen in CL1-0 cells should be attributed to its higher sensitivity to terpinen-4-ol, thereby causing DNA fragmentation to occur earlier. Taken together, these results suggest that terpinen-4-ol induces apoptosis in A549 and CL1-0 cells.

### 3.3. Terpinen-4-ol Induces Caspase-Dependent Cell Death in A549 and CL1-0 Cells

**Figure 3:**
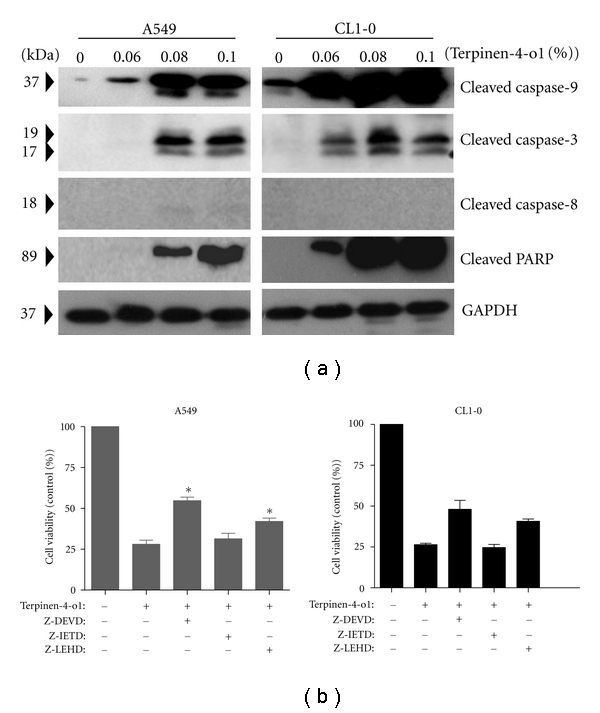
Effects of terpinen-4-ol on caspase activation in A549 and CL1-0 cells. (a) Cells were treated with the indicated concentrations of terpinen-4-ol for 24 hours. Total cell lysates were prepared, resolved by SDS-PAGE, and immunoblotted with the indicated antibodies to detect the cleaved forms of caspase-8, caspase-9, caspase-3, and PARP. Data are representative of three independent experiments with similar results. (b) Cells were treated with terpinen-4-ol and/or the indicated caspase inhibitor for 24 hours, and cell viability was determined using the MTT assay. Data are mean ± SD of three independent experiments.

Apoptosis can be elicited by chemotherapeutic agents, and the signal transduction can be generally divided into the intrinsic, mitochondria-mediated, and extrinsic pathways [18]. These pathways converge at several downstream points, including caspase-3, and/or caspase-7. Activated caspase-3 and/or caspase-7 then cleave poly(ADP-ribose)polymerase (PARP), which eventually leads to apoptosis. Thus, to clarify the type of apoptotic pathway induced by terpinen-4-ol, the cleaved forms of caspase-8, caspase-9, caspase-3 and PARP were determined by Western blot analysis. As illustrated in Figure 3(a), the cleaved/activated forms of caspase-9, caspase-3, and PARP, but not caspase-8, were induced at higher concentrations of terpinen-4-ol in A549 and CL1-0 cells. Activation of caspase-9 and caspase-3 by terpinen-4-ol suggests that the mitochondrial pathway is involved. Therefore, we used various caspase inhibitors to investigate one step further. As illustrated in Figure 3(b), the specific caspase 8 inhibitor Z-IETD was completely ineffective at increasing cell viability, thereby excluding the possibility of involvement of the extrinsic pathway in terpinen-4-ol-induced apoptosis. However, caspase-3 inhibitor Z-DEVD and the caspase-9 inhibitor Z-LEHD were effective in rescuing both A549 and CL1-0 cells, indicating that the intrinsic, mitochondria-mediated apoptotic pathway was the mechanism executing terpinen-4-ol-induced apoptosis.

### 3.4. Terpinen-4-ol Induced Loss of Mitochondrial Membrane Potential and Cytochrome C Release

**Figure 4:**
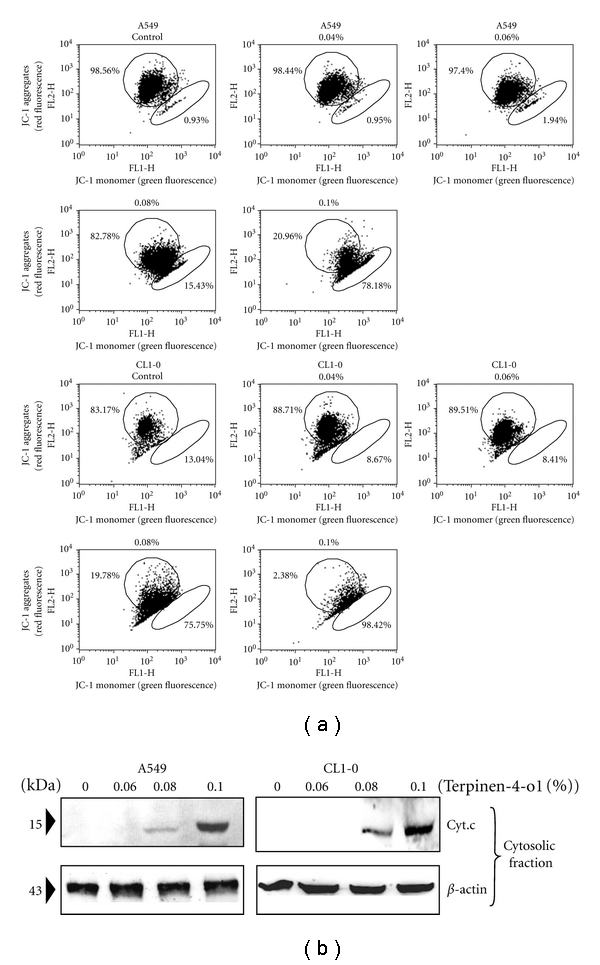
Effects of terpinen-4-ol on mitochondrial membrane potential and cytochrome c release in A549 and CL1-0 cells. (a) The cells were stained with JC-1 fluorescence dye and the change in mitochondrial membrane potential (ΔΨ_*m*_) was examined by flow cytometry. Data are representative of three independent experiments with similar results. (b) Cytosolic lysates were prepared and subjected to SDS-PAGE followed by Western blotting with anticytochrome c antibody. Data are representative of three independent experiments showing similar results.

To confirm that the intrinsic, mitochondria-mediated apoptotic pathway was involved, we further measured the mitochondrial membrane potential (Δ*ψ*_*m*_) in terpinen-4-ol-treated A549 and CL1-0 cells. Loss of mitochondrial membrane potential Δ*ψ*_*m*_ is an indicator of mitochondrial damage during apoptosis. We used the fluorescent cationic dye JC-1 to examine the effect of terpinen-4-ol on membrane potential (Δ*ψ*_*m*_). JC-1 is a mitochondria-specific probe that aggregates in normally polarized mitochondria and emits red fluorescence. In apoptotic depolarized cells, where the mitochondrial membrane potential is reduced, JC-1 is diffused throughout the cells and assumes a monomeric form, which emits green fluorescence. A concentration-dependent decrease in red fluorescence was observed in both A549 and CL1-0 cells, suggesting that terpinen-4-ol treatment led to a reduction in Δ*ψ*_*m*_ (Figure 4(a)). A loss of Δ*ψ*_*m*_ can lead to cytochrome c release from the mitochondria into the cytosol, an important event for apoptosis induction [19]. Thus, the cytosolic fractions of A549 and CL1-0 cells treated with various doses of terpinen-4-ol were prepared and analyzed for cytochrome c release by Western blotting. As shown in Figure 4(b), cytochrome c expression in the cytosolic fractions was observed in both A549 and CL1-0 cells treated with higher concentrations of terpinen-4-ol. Taken together, we verified that the mitochondrial pathway was responsible for the terpinen-4-ol-induced apoptosis.

### 3.5. Terpinen-4-ol Reduced Bcl-2, Bcl-xl and Iaps Protein Expression in A549 and CL1-0 Cells

**Figure 5:**
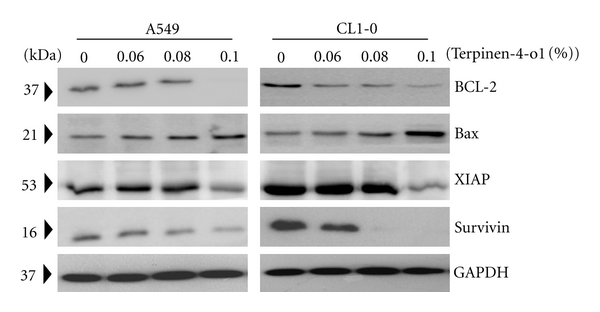
Effects of terpinen-4-ol on the expression of Bcl-2 family proteins and IAPs in A549 and CL1-0 cells. Cells were treated with the indicated concentrations of terpinen-4-ol for 24 hours. Total cell lysates were prepared, subjected to SDS-PAGE, and immunoblotted with antibodies to detect Bcl-2, Bcl-xl, Bax, XIAP, and survivin. Data are representative of three independent experiments with similar results.

Having shown mitochondrial involvement in terpinen-4-ol-induced apoptosis, we next focused on the Bcl-2 family members, which are known regulators of cytochrome c release from mitochondria during apoptosis. We examined the protein expression of a proapoptotic member, Bax, which triggers cytochrome c release, and an antiapoptotic member, Bcl-2, which inhibits cytochrome c release [20]. Higher doses of terpinen-4-ol treatment on A549 and CL1-0 cells for 24 hours resulted in decreased Bcl-2 protein levels and increased Bax protein expression (Figure 5). In addition to the Bcl-2 family, the IAP family proteins also regulate caspase activity, thus affecting apoptosis [21]. Therefore, the expression of two IAP family proteins, survivin and XIAP, were determined. As shown in Figure 5, XIAP and survivin protein expression were reduced at higher doses of terpinen-4-ol.

### 3.6. Terpinen-4-ol-Induced Apoptosis Is p53 Dependent

**Figure 6:**
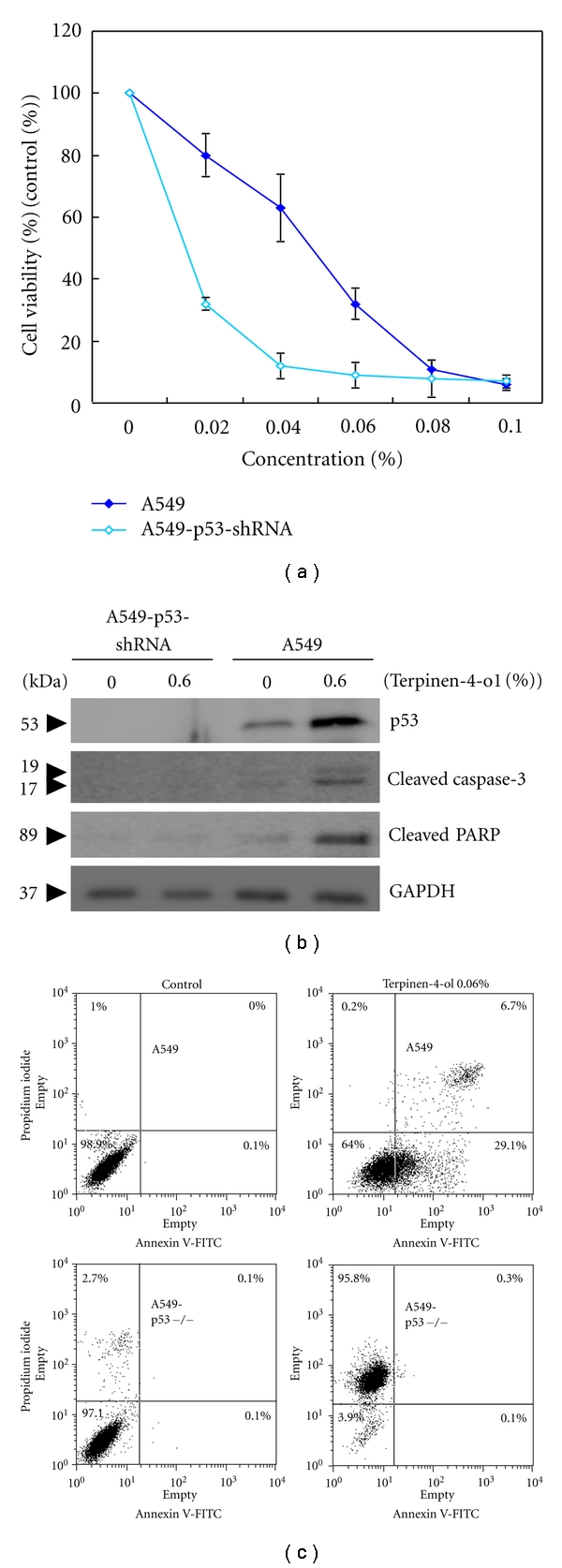
Effect of terpinen-4-ol on cell viability, caspase 3 activation, and PARP cleavage in A549 and p53-silenced A549 cells. (a) A549 and p53-silenced A549 cells were seeded at 5 × 10^4^ cells/well and then treated with the indicated concentrations of terpinen-4-ol for 24 hours following attachment. Cell viability was determined using the MTT assay. Each point on the graph represents the mean ± SD of triplicate tests. *P value <.05 compared with the untreated control group. (b) A549 and p53-silenced A549 cells were treated with the indicated concentrations of terpinen-4-ol for 24 hours. Total cell lysates were prepared, resolved by SDS-PAGE and immunoblotted with the indicated antibodies to detect the cleaved forms of caspase-3 and PARP. Data are representative of three independent experiments with similar results. (c) A549 and p53-silenced A549 cells were treated with 0 or 0.06% terpinen-4-ol for 6 hours and subjected to flow cytometry analysis after staining with annexin V-FITC and propidium iodide (PI). The lower right quadrant (annexin V+/PI−) represents early apoptosis, and the upper right quadrant (annexin V+/PI+) represents late apoptosis and necrosis. Data are a representative of three independent experiments showing similar results.

p53 is known to play important roles in apoptosis [22, 23]; therefore, we wanted to determine whether p53 plays an important role in terpinen-4-ol-induced apoptosis. Our results showed that tepinen-4-ol increased p53 protein expression in both A549 and CL1-0 cells. To further clarify the role of p53 protein, p53 was diminished by shRNA in A549 cells. Although cell viability, as determined by the MTT assay following terpinen-4-ol treatment, was significantly lower in p53-diminished A549 cells compared with wild-type A549 cells (Figure 6(a)), we surprisingly found that terpinen-4-ol induced mostly necrosis in these cells based on annexin V flow cytometric analyses (Figure 6(c)). The necrotic cell death was characterized as PI+/annexin V-by flow cytometry. Further analysis demonstrated that terpinen-4-ol failed to activate caspase 3 and PARP in p53-deficient A549 cells (Figure 6(b)), which confirmed that necrotic cell death was occurring. These results suggest that p53 is a prerequisite to terpinen-4-ol-induced apoptosis.

### 3.7. Terpinen-4-ol Inhibits A549 Tumor Growth in a S.C. Tumor Model

**Figure 7:**
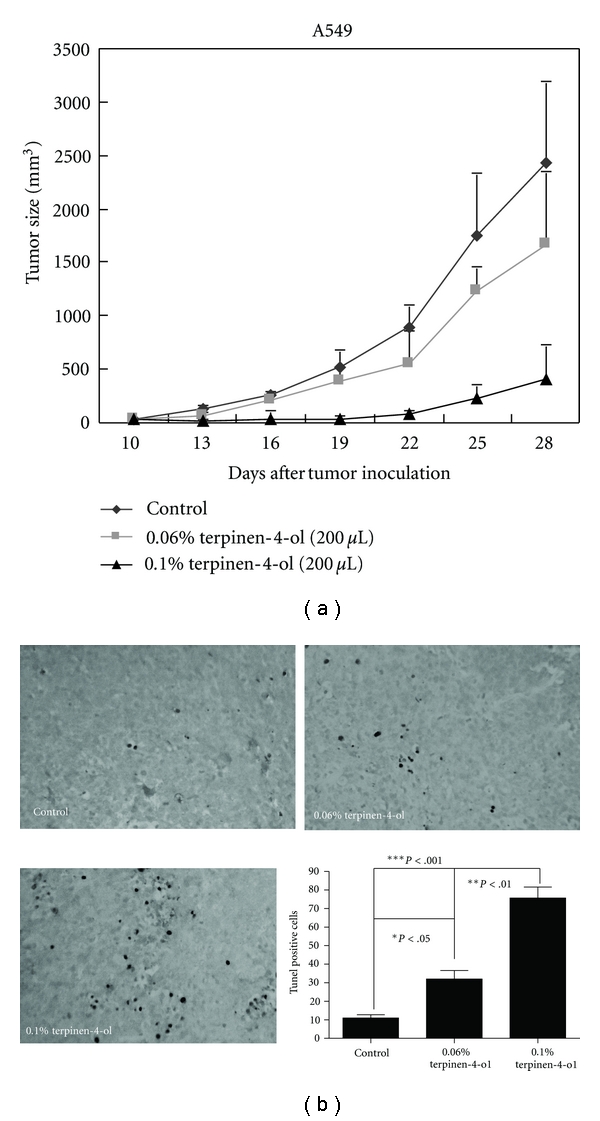
Effects of terpinen-4-ol on tumor growth in vivo. (a) 1 × 10^7^ A549 cells were s.c. injected into the right flank of BALB/*c nu/nu* mice (*n* = 5), followed by terpinen-4-ol therapy, as described in the Materials and Methods. The mean tumor volume was measured every three days from the onset of terpinen-4-ol treatment and plotted. Experiments were repeated two times and provided similar results. (b) TUNEL labeling of apoptotic DNA fragmentation in A549 tumor sections from PBS-treated control animals and animals treated with terpinen-4-ol. Apoptotic cells were identified as dark brown nuclei using light microscopy. The number of apoptotic cells was counted under high magnification (400×) in five randomly chosen fields for each sample. The data are presented as the mean ± SD of groups of three samples pooled from two independent experiments.

The prominent inhibitory effect of terpinen-4-ol on NSCLC cell proliferation in vitro suggested that it might suppress tumor growth *in vivo*. To verify this hypothesis, female BALB/*c nu/nu* mice were s.c. inoculated with A549 cells, followed by the indicated doses of terpinen-4-ol therapy starting on day 10 after tumor inoculation. As shown in Figure 7(a), terpinen-4-ol dose-dependently reduced tumor volume in BALB/*c nu/nu* mice. To further confirm the ability of terpinen-4-ol to elicit apoptosis in vivo, in situ TUNEL staining was carried out on tissue sections of tumors excised on day 28 from A549 cell-implanted mice treated with various doses of terpinen-4-ol. As illustrated in Figure 7(b), 0.1% terpinen-4-ol treatment caused a significantly higher percentage of TUNEL-positive apoptotic cells compared with vehicle treatment. These results indicated that terpinen-4-ol also inhibits the growth of nonsmall lung cancer cells and is a potent apoptosis-inducing agent in vivo.

## 4. Discussion

Terpinen-4-ol, a major component of essential oil from several aromatic plants, is used as an anti-inflammatory and antioxidant agent [9–13]. This compound also exhibits antiproliferative and antitumor effects in the murine AE17 mesothelioma and B16 melanoma tumor cell lines and human melanoma M14 WT cells, as well as their drug-resistant counterparts, M14 adriamicin-resistant cells [14, 15]. Our results demonstrate for the first time that terpinen-4-ol induces apoptosis in human lung cancer cells in vitro and in vivo. Elevation of the Bax/Bcl-2 ratio and a decrease in IAP family proteins XIAP and survivin were observed following terpinen-4-ol treatment. Notably, our results also indicated that terpinen-4-ol-induced apoptosis is p53 dependent. 

Apoptosis and necrosis are two typical types of cell death [18]. Apoptosis is characterized by several biochemical criteria such as changes in mitochondrial membrane permeability, caspase signaling activation, internucleosomal DNA cleavage, and the release of intermembrane mitochondrial proteins [24]. In contrast, necrosis is characterized mostly in negative terms by the absence of apoptosis parameters, such as caspase activation [25]. Recently, accumulating evidence has indicated that the dysregulation of apoptosis contributes to carcinogenesis [26, 27]. Therefore, it has been suggested that susceptibility to chemotherapeutic agents is dependent on the ability of tumor cells to respond to apoptosis. Terpinen-4-ol has been shown to induce caspase-dependent apoptosis in human melanoma cells. However, terpinen-4-ol has also been shown to induce necrosis in AE17 murine mesothelioma and B16 murine melanoma cells [14]. The difference in type of cell death elicited by terpinen-4-ol might be explained by species or cell type differences. In our present study, the presence of early apoptotic cells (annexin V+/PI−), activated forms of caspase-9 and caspase-3, and PARP cleavage indicated that apoptosis, rather than necrosis, was involved in terpinen-4-ol-induced A549 and CL1-0 cell death (Figures 2 and 3). Furthermore, the decrease in mitochondrial membrane potential and increase in cytochrome c release into the cytosol confirmed involvement of the mitochondrial pathway of apoptosis upon the treatment with terpinen-4-ol. 

Bcl-2 family proteins including proteins that suppress apoptosis, such as Bcl-2, Bcl-X_L_, and Mcl-1, and proteins that promote apoptosis, such as Bad, Bax, and Bid, have been reported to play a central role in regulating cytochrome c release from mitochondria [20]. Thus, the balance between the expression levels of antiapoptotic and proapoptotic Bcl-2 family proteins is critical for the fate of a cell. Moreover, IAPs have also been reported to inhibit apoptosis by functioning as inhibitors of activated effector caspases, such as caspase-3 and caspase-7. IAPs are also able to inhibit the cytochrome c-induced activation of caspase-9 [21]. Our data revealed that the protein expression of antiapoptotic Bcl-2, XIAP, and survivin were reduced and that of proapoptotic Bax was elevated 24 hours after terpinen-4-ol treatment (Figure 4). These results revealed the molecular events occurring during terpinen-4-ol-induced mitochondria-mediated apoptosis.

The p53 tumor-suppressor protein protects against cancer by regulating the cellular response to DNA damage, apoptosis, and oncogene activation [22, 23]. Diminution of p53 expression by shRNA did not decrease the susceptibility of A549 cells to terpinen-4-ol-induced cytotoxicity, but a significant reduction in the proportion of apoptosis was observed in p53-diminished A549 cells. Instead of apoptosis, necrosis manifested as a result of p53 diminution in A549 cells (Figure 6). This finding suggests that p53 is essential to terpinen-4-ol-induced apoptosis in human NSCLC cancer cells and that necrosis is responsible for the cytotoxic effect of terpinen-4-ol in p53-null NSCLC cancer cells. Therefore, the presence of functional p53 acts as the key factor in switching necrosis to apoptosis in human NSCLC. This is similar to a previous study from Lim et al. in which ursodeoxycholic acid was able to switch oxaliplatin-induced necrosis to apoptosis by activating the p53-caspase 8 pathway in HepG2 hepatocellular carcinoma [28]. Our results do not exclude the possibility of a failure to induce apoptosis in cancer cells, where p53 is mutated and loses its transactivation ability, because p53 mutations are varied. In other words, terpinen-4-ol is most effective against cancers with functional p53.

Cell-cycle arrest is a common cause of cell growth inhibition [29]. Previous studies have indicated that treatment with terpinen-4-ol induces antiproliferative effects by causing G_1_ phase arrest in AE17 murine mesothelioma and B16 murine melanoma cells [14]. However, in the present study, we observed that the exposure of A549 and CL1-0 cells to terpinen-4-ol caused G_2_/M phase arrest (Figure 2(a)). Again, the difference in type of cell-cycle arrest elicited by terpinen-4-ol might be due to species or cell-type differences. Cell-cycle progression is controlled by the activation of a highly conserved family of protein kinases, the cyclin-dependent kinases (Cdks), and the expression of their cyclin partners, whereas inhibitors of Cdks, including p21 and p27, inhibit Cdk activity by binding to Cdk-cyclin complexes [30, 31]. Assembly of the cyclin B1/Cdc2 protein complex is essential for G_2_-phase progression and G_2_/M transition [32]. In addition Cdc25c phosphatase is a key activator of Cdc2/cyclin B at the G_2_/M transition. Cdc25c functions by dephosphorylating, and thereby activating, Cdc2 [33]. Although it is not fully understood how DNA damage causes cells to undergo apoptosis at the G_2_/M checkpoint, our results indicate that the expression of cyclin B1 and cdc25c decrease when cells are treated with terpinen-4-ol as compared to the untreated control (see in supplementary materials in doi: 10.1155/2012/818261). However, the expression of Cdc2 and cdk inhibitors p21 and p27 was unaffected by terpinen-4-ol (see in supplementary materials in doi: 10.1155/2012/818261). One of the important mechanisms through which p53 can regulate the cell cycle is to induce cdk inhibitor p21, which can induce G_2_/M or G1 arrest [34]. Because p21 and p27 protein expression remained unchanged following terpinen-4-ol treatment, it is plausible that terpinen-4-ol induces G_2_/M arrest via a p53 independent mechanism, and that decreases in the expression of cyclin B1 and Cdc25c contribute mainly to the G_2_/M arrest seen in terpinen-4-ol-treated lung cancer cells.

In conclusion, we demonstrated that caspase-dependent mitochondrial dysfunction is the mechanism of terpinen-4-ol-induced apoptosis in nonsmall lung cancer cells. Downregulation of Bcl-2, XIAP and survivin suggests that terpinen-4-ol increases the susceptibility of NSCLC cells to apoptosis induction. Notably, the ability of terpinen-4-ol to induce apoptosis in NSCLC cells is p53-dependent. Finally, our data also showed that the growth of s.c. xenograft tumors was remarkably inhibited by intratumoral injection of terpinen-4-ol, indicating that the agent also has potential for clinical anticancer activity. However, further work is needed to develop a suitable formulation for either systemic administration or convection-enhanced delivery of the compound to patients.

## Supplementary Material

Effects of terpinen-4-ol on the expression of cell cycle-related proteins in A549 and CL1-0 cells. Cells were treated with indicated concentrations of terpinen-4-ol for 24 hr. Total cell lysates prepared were subjected to SDS-PAGE and immunoblotted with antibodies to detect cyclin B1, phospho-cdc2 (Tyr15), Cdc25, p27 and p21 protein expressions. Data correspond to a representative of three independent experiments with similar results.
