# Supplementary material for: Terpinen-4-ol Induces Apoptosis in Human Nonsmall Cell Lung Cancer In Vitro and In Vivo
Source: Evid Based Complement Alternat Med. 2011 Jun 20;2012:818261. doi: 10.1155/2012/818261 (PMC3133878; doi:10.1155/2012/818261)
Supplement: Supplementary file 1 — Effects of terpinen-4-ol on the expression of cell cycle-related proteins in A549 and CL1-0 cells. Cells were treated with indicated concentrations of terpinen-4-ol for 24 hr. Total cell lysates prepared were subjected to SDS-PAGE and immunoblotted with antibodies to detect cyclin B1, phospho-cdc2 (Tyr15), Cdc25, p27 and p21 protein expressions. Data correspond to a representative of three independent experiments with similar results. [file 818261.f1.ppt]

## Slide 1
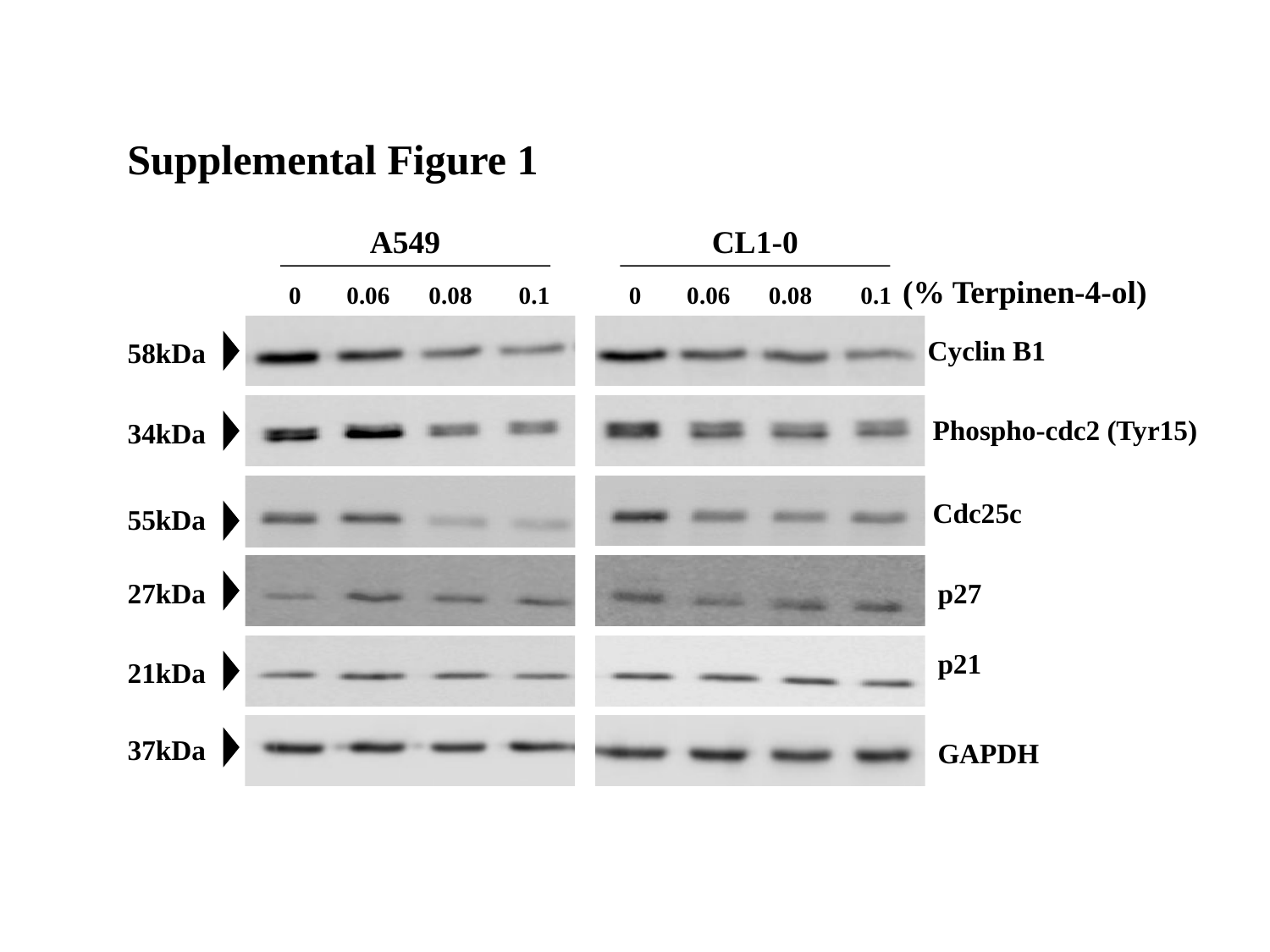

Supplemental Figure 1
A549
CL1-0
(% Terpinen-4-ol)
 0
 0.06
 0.08
 0.1
 0
 0.06
 0.08
 0.1
Cyclin B1
58kDa
Phospho-cdc2 (Tyr15)
34kDa
Cdc25c
55kDa
27kDa
p27
p21
21kDa
37kDa
GAPDH
